# Initial Rotational Instability of the Tapered Wedge-Shaped Type Cementless Stem

**DOI:** 10.1155/2020/2180260

**Published:** 2020-09-22

**Authors:** Yosuke Iwamoto, Hiroaki Kijima, Hiroshi Tazawa, Natsuo Konishi, Hitoshi Kubota, Shin Yamada, Takayuki Tani, Keiji Kamo, Norio Suzuki, Yoshihiko Okudera, Masashi Fujii, Ken Sasaki, Tetsuya Kawano, Itsuki Nagahata, Naohisa Miyakoshi, Yoichi Shimada

**Affiliations:** ^1^Department of Orthopedic Surgery, Akita University Graduate School of Medicine, 1-1-1 Hondo, Akita 010-8543, Japan; ^2^Akita Hip Research Group, 1-1-1 Hondo, Akita 010-8543, Japan

## Abstract

**Background:**

Because the tapered wedge-shaped type cementless stem has a small anteroposterior width and a low occupation rate in the medullary space, postoperative rotational instability and stem subsidence due to inadequate proximal fixation are concerns. The purpose of this study was to clarify the relationship between the rotational instability of the tapered wedge-shaped type cementless stem and femoral canal shape.

**Methods:**

A total of 61 primary total hip arthroplasties with the tapered wedge-shaped type cementless stem Accolade® TMZF (11 males, 50 females; mean age 60 years) from January 2012 to June 2015 who underwent computed tomography before surgery and within 4 weeks and 1 year after surgery were evaluated. The preoperative femoral neck anteversion angle, preoperative femoral canal flair index, the degree of postoperative stem subsidence within 1 year after operation, and the degree of rotational change in the stem setting angle within 1 year after operation were investigated.

**Results:**

The mean preoperative femoral neck anteversion angle was 32.2° ± 17.8° (0°–69°), and the mean preoperative canal flair index was 3.68 ± 0.58 (2.44–5.55). There were no stem subsidence cases within 1 year after operation. The mean degree of rotational change in the stem from immediately to 1 year after surgery was −0.4° ± 1.7° (−3°–3°). There was no significant correlation between the canal flair index and the rotational change in the stem. In addition, the mean difference between the preoperative femoral neck anteversion angle and the stem rotational angle immediately after surgery was only 1.3° ± 5.3° (−29°–15°).

**Conclusions:**

In all cases, including stove-pipe cases, the degree of rotational change in the Accolade® TMZF stem from immediately to 1 year after surgery was within 3°. In other words, regardless of femoral canal shape, the tapered wedge-shaped type cementless stem has little initial rotational instability.

## 1. Introduction

The tapered wedge-shaped type cementless stem is based on the concept of mediolateral fixation of the metaphysis. There has been a report that its initial fixation is good because it is resistant to settling and rotation by being pressed while compressing the surrounding cancellous bone [1]. Other reports indicated that it is possible to preserve cancellous bone because the shoulder part of such a stem is reduced, the anteroposterior width is narrow, and there are few perioperative complications such as intraoperative fracture [2, 3]. In addition, progression of proximal femoral atrophy due to stress shielding is considered mild because load is applied proximally to the femur due to the shorter length of the stem and the distal taper to block excessive distal fixation [4]. On the contrary, when the tapered wedge-shaped type cementless stem is inserted, for example, to stove-pipe type femoral canal cases, there is a fear of postoperative rotational instability and stem subsidence due to inadequate proximal fixation because its anteroposterior width is small, and the medullary space occupancy rate of such stems is low [5].

However, there has been no report of a detailed investigation of the initial rotational instability of the tapered wedge-shaped type cementless stem. In order to evaluate the rotational angle of the stem setting in detail, it is impossible to evaluate it only by radiography; it is necessary to evaluate it using axial images of computed tomography (CT). By evaluating stem rotational instability in detail using CT, the initial rotational instability of such stems, for example, for stove-pipe-type femurs due to osteoporosis, can be compared with cases in which such stems are used for canal-type femurs. In other words, by evaluating the degree of rotational change in the stem setting angle after surgery, it is possible to clarify the condition in which rotational instability of the tapered wedge-shaped type cementless stem is likely to occur, and it becomes possible to clarify the criteria for using such stems. Therefore, the purpose of this study was to clarify the relationship between rotational instability of the tapered wedge-shaped type cementless stem and the femoral canal shape type.

## 2. Materials and Methods

A total of 61 primary total hip arthroplasty (THA) cases with the tapered wedge-shaped type cementless stem Accolade® TMZF (Stryker Orthopaedics, Mahwah, New Jersey, USA) from January 2012 to June 2015 in a single hospital and who underwent CT (Revolution CT, Cytiva, Tokyo, Japan) before, within 4 weeks, and 1 year after surgery were evaluated. Thus, this is the retrospective study.

And all surgeries were operated by a single surgeon, and all surgical approaches used the direct lateral approach. The patients' average age was 60 years (range, 32–80 years); 11 were male, and 50 were female. The mean follow-up period was 2.7 years (13–44 months).

The preoperative femoral neck anteversion angle, preoperative femoral canal flair index (CFI), the degree of postoperative stem subsidence within 1 year after surgery, and the degree of rotational change in the stem setting angle within 1 year after surgery were investigated.

**Figure 1:**
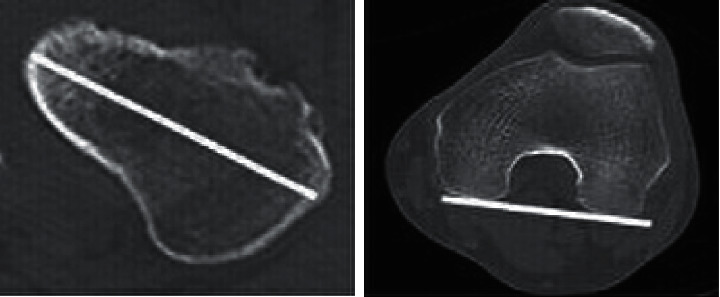
Femoral neck anteversion angle. The femoral neck anteversion angle is measured by identifying the femoral head-neck axis from the CT horizontal section slice just beneath the femoral head, with reference to the posterior condylar axis.

The preoperative femoral neck anteversion angle was the angle on the axial CT images from the line connecting the posterior projecting point of the distal medial and lateral condyles of the femur (posterior condylar axis) to the line of the femoral head-neck axis just beneath the femoral head (Figure 1) [6]. Preoperative medullary canal shape was evaluated using the canal-flare index (CFI). [7] Stem subsidence was judged on X-ray by whether the stem 1 year after operation moved distally more than 2 mm compared with the position of the stem on X-ray immediately after surgery.

**Figure 2:**
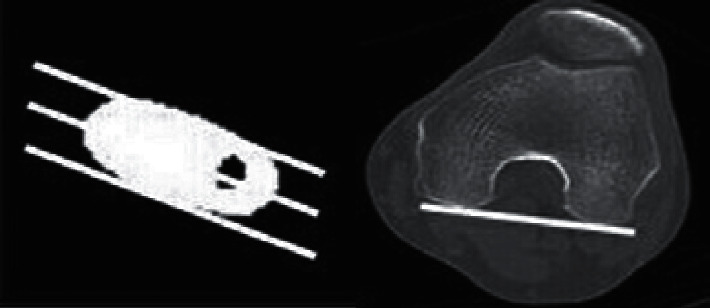
Stem rotation setting angle. The stem rotation setting angle is measured using the rotation axis of the stem from the anterior/posterior stem in the CT horizontal section with reference to the posterior condylar axis.

The stem rotational setting angle was measured as the axis of the anterior/posterior stem on axial CT images with reference to the posterior condylar axis, and the degree of rotational change in the stem setting angle immediately to 1 year after surgery was evaluated (Figure 2) [8].

In addition, clinical outcomes were evaluated by the Japanese Orthopaedic Association Hip Score (JOA score) [9] to quantify hip function and by the Japanese Orthopaedic Association Hip Disease Evaluation Questionnaire (JHEQ) [10] to evaluate patient-reported outcomes.

The patients and their families were informed that data from the case would be submitted for publication and gave their consent. Patients and their families were also informed and agreed to have a CT scan before and immediately after surgery and one year after surgery and the associated increase in radiation dose. Approval was given by the institutional review board.

## 3. Results

The mean preoperative femoral neck anteversion angle was 32.2° ± 17.8° (0°–69°), and the mean preoperative CFI was 3.68 ± 0.58 (2.44–5.55). There were 2 champagne-flute cases (CFI > 4.7), 7 stove-pipe cases (CFI < 3), and 52 normal canal-type cases.

There were no cases of stem subsidence within 1 year after surgery. The mean stem rotational angle was 30.7° ± 16.1° (−6°–60°) immediately after surgery and 30.1° ± 16.2° (−8°–61°) 1 year after surgery. The mean degree of rotational change in the stem from immediately to 1 year after surgery was −0.4° ± 1.7° (−3°–3°).

**Figure 3:**
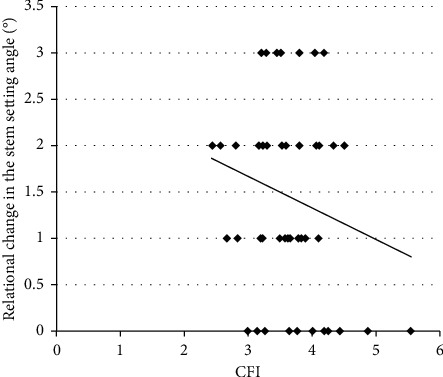
Correlation between the CFI and rotational change in the stem setting angle. There is no significant correlation between the CFI and rotational change in the stem setting angle (*r* = −0.21, *p*=0.2338, Spearman's rank correlation coefficient).

**Figure 4:**
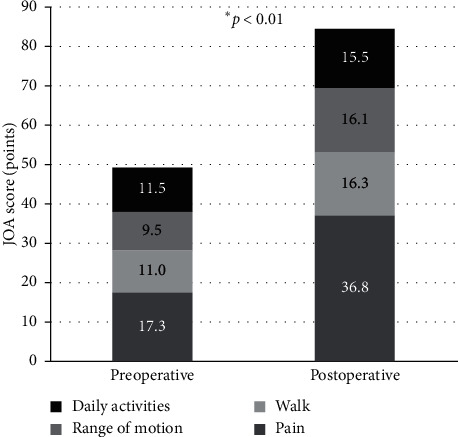
Preoperative and postoperative JOA scores. The JOA score is significantly improved from the preoperative mean of 49.3 to the postoperative mean of 84.7 (*p*=0.7336, Wilcoxon rank sum test).

**Figure 5:**
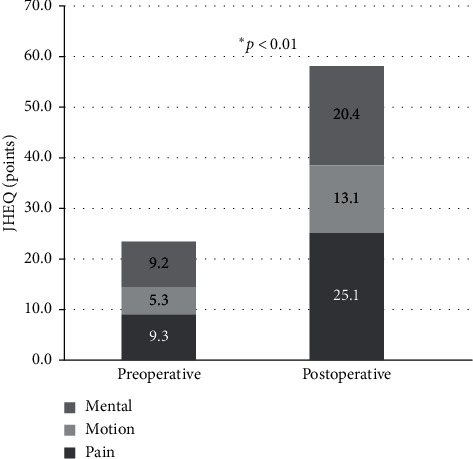
Preoperative and postoperative JHEQ scores. The JHEQ is significantly improved from the preoperative mean of 23.8 to the postoperative mean of 58.6 (*p*=0.6890, Wilcoxon rank sum test).

There was no significant correlation between the CFI and rotational change in the stem setting angle (*r* = −0.21, *p*=0.2338, Spearman's rank correlation coefficient) (Figure 3). In addition, the mean difference between the preoperative femoral neck anteversion angle and the stem rotational angle immediately after surgery was only 1.3° ± 5.3°. In almost all cases, the stem was set at the same angle as the preoperative femoral neck anteversion angle. The JOA and JHEQ scores were significantly improved from preoperative (49.3 and 23.8, respectively) to postoperative (84.7 and 58.6) (all *p* < 0.0001, Wilcoxon rank sum test) (Figures 4 and 5).

There was no correlation between the improvement rate of the JOA score and rotational change in the stem setting angle (*r* = 0.01 *p*=0.7336, Spearman's rank correlation coefficient) and no significant correlation between the JHEQ score improvement rate and rotational change in the stem setting angle *(r* = −0.01 *p*=0.6890, Spearman's rank correlation coefficient).

## 4. Discussion

Few reports have evaluated the rotational angle of the stem with the passage of time from immediately to 1 year after surgery. Evaluation of the change in the stem rotational angle in detail using the axial CT images in the present study showed that the tapered wedge-shaped type cementless stem maintained rotational stability irrespective of the canal shape.

There have been many reports of good middle-to-long clinical outcomes of tapered wedge-shaped type cementless stems [1, 11]. Thus, this stem type is one of the most commonly used types of cementless stems at present.

However, there are reports of stem sinking and rotated dislocation cases [2] or of intraoperative femoral fracture cases that were thought to be the result of attempting to achieve more stable stem setting [3]. It has also been suggested that one cause of this instability is the rotational instability due to the small anteroposterior width and the low occupation rate in the medullary space. However, the rotational change in the stem setting angle at 1 year after surgery of Accolade® TMZF was very small in all cases, including stove-pipe cases, in the present study.

In the case studied in present research, the stems were inserted at the same anteversion angle of the femoral neck, and no effort has been made to change the rotation angle of the stem to a different anteversion angle than the patient's femoral neck. As a result, the stem fits in the area with the widest mediolateral widths; thus, the rotational instability did not occur, no matter how small the anteroposterior width of the stems was.

One of the limitations of this study is the accuracy of measurement on CT images. In this study, measurements of the femoral neck anteversion anterior angle and the stem rotation setting angle were performed using axial CT images. Measurement error is considered to occur due to differences in limb positioning during CT imaging [10]. It has also been reported that the femoral neck anteversion angle varies depending on the slice by a few millimetres [8]. However, Sugano et al. [8] reported no significant difference in the difference of the femoral neck axis taken by 3-dimensional CT although the mean measurement difference is only 1.4°, and the femoral neck axis measured with the slice just below the femoral head does not differ significantly [8]. Since the stem rotational angle was measured in the present study, it could be measured more accurately than femoral neck anteversion.

Current study did not take into account the patient's physique and the effects of postoperative rehabilitation. These were also the limitation of this study. Although there were no significant obesity cases included in this study, it is undeniable that allowing full weight-bearing early in the postoperative period may cause rotational instability in such cases.

Other limitations are that all cases were only from a single hospital, and the number of cases was small. Because the surgical technique was almost constant without performing intraoperative version control in the present study, the stem was set at the same angle in the femoral neck anteversion angle in almost all cases. If a similar investigation were to be conducted at other facilities in which the surgeons perform a “stem intraoperative version control procedure” under another concept or if similar investigations were to be performed with larger numbers of cases with the same procedure, more cases with rotational instability might be found. Identifying such cases will lead to the establishment of better indication criteria and stem setting technique of tapered wedge-shaped type cementless stems. This study provides the first step to that end.

## 5. Conclusion

In all cases, including stove-pipe canal cases, the rotational change in the Accolade® TMZF stem from immediately to 1 year after surgery was within 3°. Thus, the tapered wedge-shaped type cementless stem has little initial rotational instability, regardless of femoral canal shape type.

## Data Availability

All data generated or analysed during this study are included in this published article.
